# Online Search Volume Related to Ulnar Collateral Ligament Repair Has Increased More Rapidly Than Reconstruction Over the Past Decade: A Google Trends Analysis

**DOI:** 10.1002/ars2.70076

**Published:** 2026-09-12

**Authors:** Benjamin W. King, S. Brody Westbrooks, Evan P. Bailey, Jesse Seilern Und Aspang, David Marvin, Kyle Hammond, Richard M. Danilkowicz

**Affiliations:** ^1^ Emory University School of Medicine Atlanta Georgia U.S.A.; ^2^ Mercer University School of Medicine Savannah Georgia U.S.A.

## Abstract

**Purpose:**

To evaluate online public interest in ulnar collateral ligament (UCL) reconstruction and repair from 2015 to 2025 using Google Trends.

**Methods:**

Relative search volume (RSV) data from searches related to UCL reconstruction and repair from January 2015 to December 2025 were obtained from Google Trends. The 3 search terms related to each procedure with the highest RSV during the study period were included, and individual search term data was averaged for each procedure for analysis. Statistical analysis included linear regression to evaluate changes in RSV over time and analysis of covariance to compare the slopes of the regression lines between UCL reconstruction and UCL repair.

**Results:**

Linear regression showed that public interest in UCL repair increased significantly from 2015 to 2025 (*R* ^2^ = 0.68; *P* = 1.3 × 10^−34^), while interest in reconstruction showed a more modest but statistically significant increase (*R* ^2^ = 0.16; *P* = 1.8 × 10^−6^). Analysis of covariance confirmed that the slope of the UCL repair 10‐year trend (*β* = 0.387) was significantly greater than that of UCL reconstruction (*β* = 0.120; *P* = 6.1 × 10^−7^). The percentage increase in mean RSV from 2015 to 2025 was 687% for UCL repair and 87.5% for UCL reconstruction.

**Conclusions:**

Online search volumes related to UCL repair have increased dramatically over the last decade, far outpacing the growth in online search volumes related to UCL reconstruction.

**Clinical Relevance:**

Given the rapid rise in online interest for UCL repair, the development of high‐quality online educational resources may be warranted to ensure that patients receive reliable and evidence‐based information regarding surgical options for UCL injuries.

## Graphical abstract

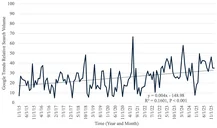

The excessive valgus stress experienced by the elbow during the overhead throwing motion places the medial ulnar collateral ligament (UCL) of baseball players at risk of injury.^1^, ^2^, ^3^ In athletes with UCL injuries that have failed nonoperative management, UCL reconstruction has long been considered the gold standard surgical intervention.^1^, ^4^ Outcomes after UCL reconstruction are generally excellent, but the prolonged absence required for return to competition can be both frustrating and costly, with prior work reporting an average recovery cost of nearly $2 million per professional pitcher after surgery.^5^, ^6^, ^7^ This prolonged rehabilitation period has long sparked interest in alternative approaches that might allow for a faster return to competition.^8^ One of the most significant recent developments in UCL injury management is the modern UCL repair with suture augmentation technique first performed by Dugas et al. in 2013.^9^ In a comparative cohort, Dugas et al. recently reported no difference in return‐to‐sport rates or patient‐reported outcomes between repair with suture augmentation and reconstruction; however, the repair cohort was able to return to practice (6.7 vs 10.2 months) and competition (9.2 vs 13.4 months) significantly sooner.^10^ Despite its appeal to patients, repair is not universally indicated for all UCL injuries. Repair is only possible when the UCL has avulsed from the medial epicondyle or sublime tubercle without substantial intrasubstance ligamentous injury.^10^, ^11^

Given the potential for an expedited return to competition with UCL repair, patients may be increasingly interested in pursuing this procedure after an injury. As modern patients rely heavily on the Internet for health care information, online search volume can serve as a proxy for public interest in various topics.^12^ Yu et al. previously evaluated trends in social media post volume related to UCL reconstruction between 2016 and 2019 and found that posts increased each year during the study period.^13^ However, their study did not assess online interest in UCL repair and was limited to public social media posts. Several prior peer‐reviewed studies have used the publicly available online tool Google Trends (Alphabet, Mountain View, CA) to evaluate public interest in orthopaedic procedures, including comparisons of autograft options for anterior cruciate ligament reconstruction.^14^, ^15^, ^16^, ^17^ Given that prior work has shown that the quality of online educational resources related to UCL injuries is generally low, a detailed analysis of online search trends related to UCL surgery is important to assess changes in how frequently the public is accessing these resources over time.^18^, ^19^, ^20^

The purpose of this study was to evaluate the online public interest in UCL reconstruction and repair from 2015 to 2025 using Google Trends. We hypothesized that the growth in public interest in UCL repair would be greater than that of UCL reconstruction given the novelty and increasing clinical adoption of UCL repair during the study period.

## METHODS

### Data Collection

Data regarding online search volumes related to UCL reconstruction and repair from January 1, 2015, to December 31, 2025, were collected from the publicly available online resource Google Trends. Google Trends reports search volume as a relative search volume (RSV) score ranging from 0 to 100, with 100 representing peak popularity for a given term. This study was deemed to be nonhuman subject research and therefore not subject to review by the Emory University Institutional Review Board.

Google Trends was used to determine the 3 search terms for each procedure with the highest relative search volumes over the study period; these terms were included in the analysis. Search terms that were not relevant to either procedure or were not among the 3 highest relative search volumes were excluded. For UCL reconstruction, the included search terms were “ulnar collateral ligament reconstruction,” “UCL reconstruction,” and “Tommy John surgery.” For UCL repair, the included search terms were “ulnar collateral ligament repair,” “UCL repair,” and “UCL internal brace.” RSV data for all included search terms were averaged for each surgical intervention. Only data from the United States geographic region were included.

### Statistical Analysis

Linear regression was used to assess the changes in RSV over time for both UCL reconstruction and UCL repair. The mean absolute percentage error, mean absolute deviation, and mean squared deviation were calculated to confirm that the linear model provided an adequate fit for each dataset. These metrics were included solely to assess model fit and were not used for comparison. Analysis of covariance was performed to compare the slopes of the regression lines between UCL reconstruction and UCL repair. Additionally, the percentage change in RSV from 2015 to 2025 was calculated for each intervention independently.

To evaluate seasonal trends in search volume, RSV data were grouped by meteorological season (winter, December through February; spring, March through May; summer, June through August; fall, September through November). Statistical significance was set at *P* < .05. Statistical analyses were performed in Microsoft Excel (Microsoft, Redmond, WA) and R (R Foundation for Statistical Computing, Vienna, Austria).

## RESULTS

### UCL Reconstruction

**FIGURE 1:**
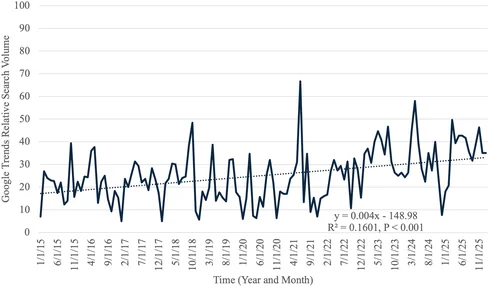
Public interest in UCL reconstruction (2015‐2025). Linear trend model including monthly relative search volume data from January 1, 2015, to December 31, 2025. (UCL, ulnar collateral ligament.)

Between 2015 and 2025, public interest in UCL reconstruction showed a statistically significant increase in the search volume, with an *R* ^2^ value of 0.16 (*P* = 1.8 × 10^−6^) (Figure 1). The UCL reconstruction linear model showed a mean absolute percentage error of 51.6, a mean absolute deviation of 8.3, and a mean squared deviation of 112.0. The percentage increase in the UCL reconstruction relative search volume from 2015 to 2025 was 87.5%.

### UCL Repair

**FIGURE 2:**
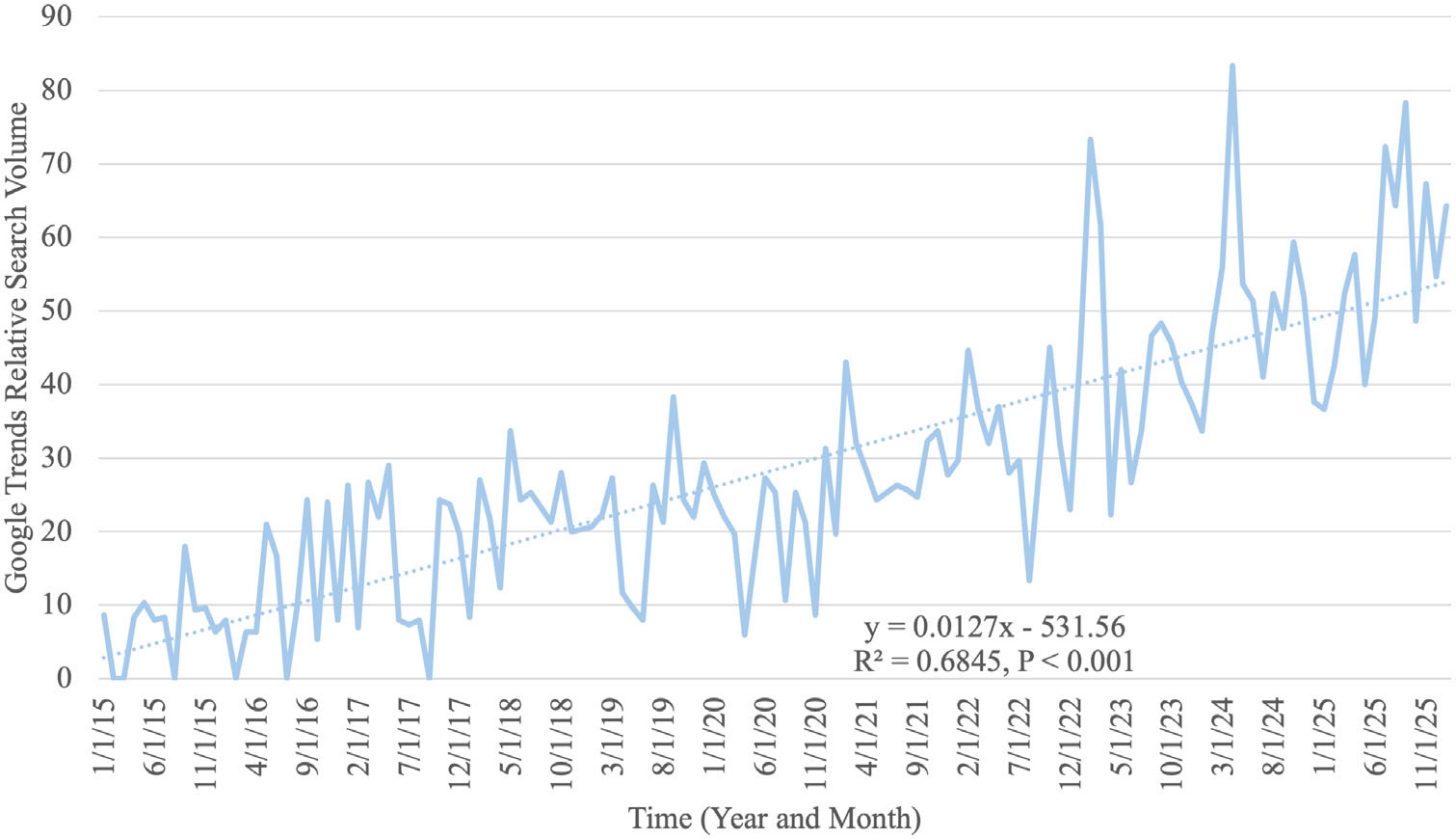
Public interest in UCL repair (2015‐2025). Linear trend model including RSV data from January 1, 2015, to December 31, 2025. (RSV, relative search volume; UCL, ulnar collateral ligament.)

**FIGURE 3:**
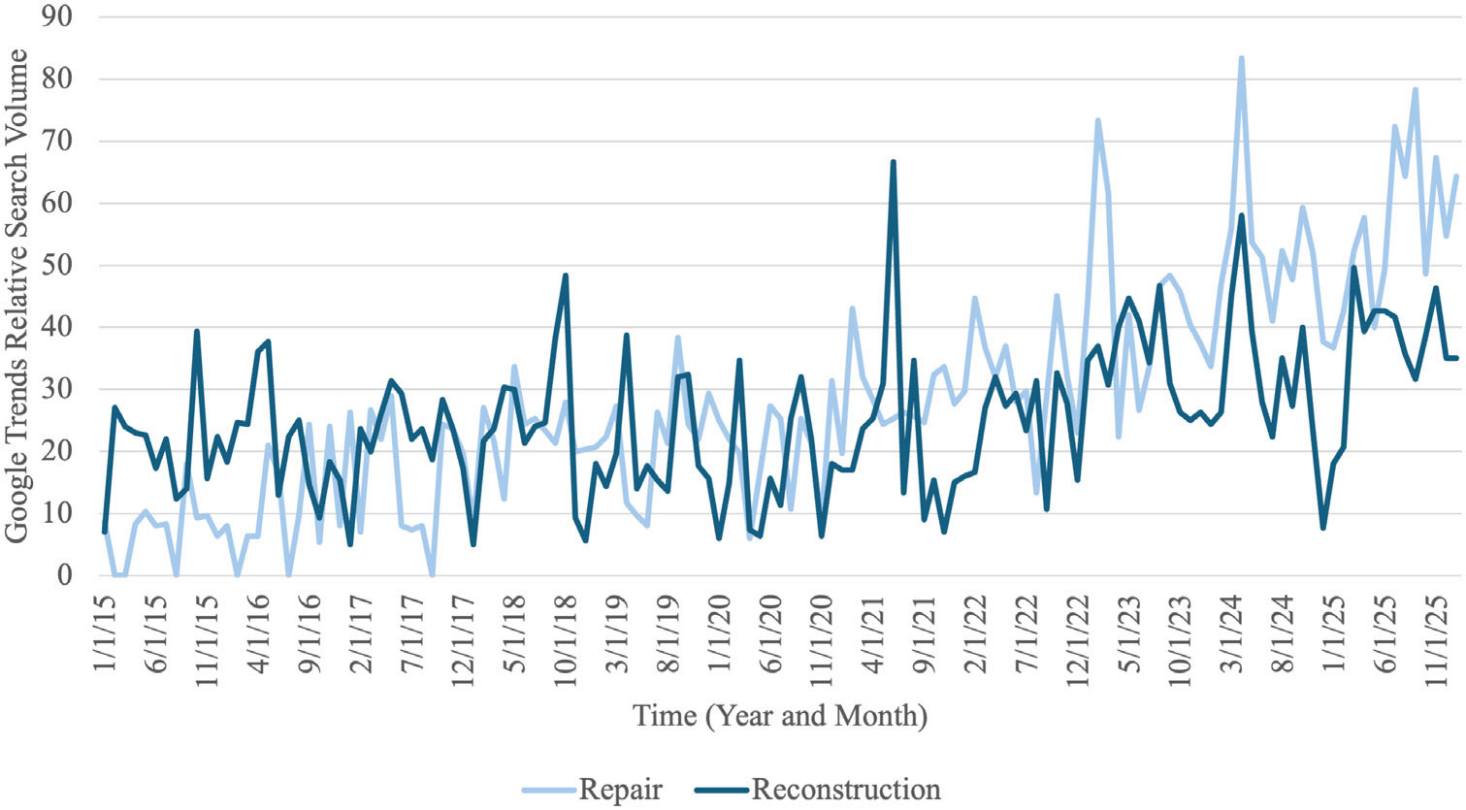
RSV changes for UCL reconstruction and repair (2015‐2025). RSV data included from January 1, 2015, to December 31, 2025. (RSV, relative search volume; UCL, ulnar collateral ligament.)

Public interest in UCL repair increased dramatically from 2015 to 2025 with an *R* ^2^ value of 0.68 (*P* = 1.3 × 10^−34^) (Figure 2). The UCL repair linear model showed a mean absolute percentage error of 39.7, a mean absolute deviation of 7.7, and a mean squared deviation of 101.9. The percentage increase in UCL repair RSV from 2015 to 2025 was 687%. On analysis of covariance testing, the UCL repair 10‐year trend slope (*β* = 0.387) was significantly greater than that of UCL reconstruction (*β* = 0.120; *P* = 6.1 × 10^−7^) (Figure 3).

### Seasonal Variation

**FIGURE 4:**
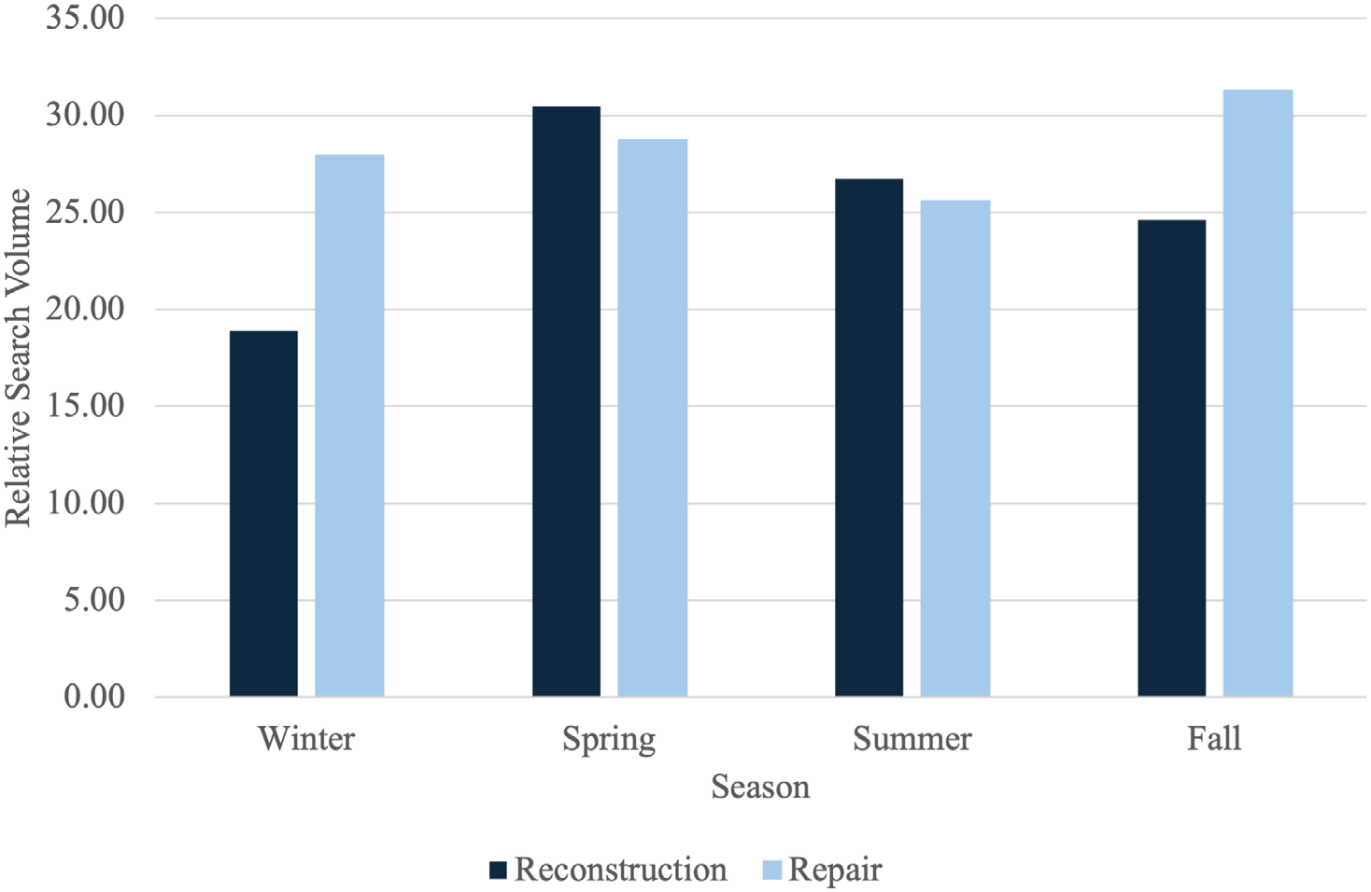
Seasonal variation in public interest in UCL reconstruction and repair. Mean relative search volume data was grouped by meteorological season (winter, December through February; spring, March through May; summer, June through August; fall, September through November). (UCL, ulnar collateral ligament.)

Public interest in UCL repair remained relatively consistent across meteorological seasons (Figure 4). Interest in UCL reconstruction was highest during spring and summer and lowest during winter.

## DISCUSSION

The most important finding of this study is that the growth of online search volume related to UCL repair markedly outpaced that of UCL reconstruction from 2015 to 2025. The relative search volume for UCL repair increased more than 680% during this period, compared with an 87.5% increase for UCL reconstruction.

In line with favorable early clinical outcomes for UCL repair, several epidemiological studies have shown that UCL repair accounts for an increasing proportion of overall UCL surgeries over time.^21^, ^22^, ^23^ Statewide database analyses in Texas and New York reported that the incidence of UCL repair rose steadily from 2010 to 2019, outpacing the growth of UCL reconstruction.^22^, ^24^ However, another study reported that UCL repair growth slowed after 2015 without explanation.^23^ These findings indicate that UCL repair is being increasingly adopted in clinical practice, reflecting broader trends in surgical management of UCL injuries.

Although epidemiologic trends show expanding clinical usage, public interest in UCL repair may be influenced more strongly by high‐profile media coverage than surgical volume. The largest spike in public interest occurred in the spring of 2023 coinciding with reports that San Francisco 49ers quarterback Brock Purdy had undergone UCL repair for an injury he sustained during the National Football League Playoffs.^25^ A second spike in early 2024 corresponded with Los Angeles Dodgers pitcher and outfielder Shohei Ohtani revealing he underwent UCL repair, noting it was “completely different” from his previous Tommy John surgery.^26^ These observations suggest that although clinical usage of UCL repair is steadily increasing, public attention fluctuates with media attention, highlighting the need for accurate and accessible patient education.

Prior research has shown that the quality of online educational resources related to UCL injuries is generally low.^18^, ^19^, ^20^ McCormick et al. previously reported that search engines return webpages specific to UCL injuries only one‐third of the time when prompted with the most commonly searched UCL injury questions.^18^ Another study evaluating the quality and comprehensibility of the most‐watched UCL‐related videos on YouTube found that nearly half were inappropriate for patient education.^27^ Prior work has reported that half of patients who use the internet for health care information share it with their physician, and approximately 30% of these patients use online information to request specific treatments.^28^ These poor quality online resources coupled with the narrow indications of UCL repair could become problematic as online public interest in UCL repair continues to grow. Reliance on inaccurate or incomplete online resources may foster unrealistic patient expectations regarding UCL repair eligibility that could ultimately strain the patient‐physician relationship. Therefore, as online public interest in UCL repair continues to rise, it is essential that physicians, professional societies, and health care organizations ensure that high‐quality, accurate online information is accessible to patients to prevent the formation of unrealistic expectations.

Physicians should be aware that the public interest in UCL repair has grown dramatically over the last decade. As this interest continues to increase, evidence‐based patient education and appropriate shared decision‐making will become increasingly important for clinicians managing UCL injuries. Further research is warranted to investigate the quality of online information specific to UCL repair and the impact that this information has on patient expectations regarding repair eligibility.

### Limitations

This study is not without limitations. First, Google Trends does not provide detailed demographic information, making it unclear whether certain populations are over‐ or under‐represented in the data. Second, the analysis includes only searches conducted through Google and does not provide any insight into the trends of other search engines. Additionally, increases in search volume may not directly reflect patient interest and could be influenced by other external factors. It is also possible that some users may use “repair” or “reconstruction” interchangeably, potentially introducing bias into keyword‐based analyses.

## CONCLUSIONS

Online search volumes related to UCL repair have increased dramatically over the last decade, far outpacing the growth in online search volumes related to UCL reconstruction.

## DISCLOSURES

The authors (B.W.K., S.B.W., E.P.B., J.S.U.A., D.M., K.H., R.M.D.) declare that they have no known competing financial interests or personal relationships that could have appeared to influence the work reported in this article.
